# Expert Consensus for Treating Cancer Patients During the Pandemic of SARS-CoV-2

**DOI:** 10.3389/fonc.2020.01555

**Published:** 2020-08-18

**Authors:** Shuang Dong, Chenggang Luo, Xuebo Hu, Jing Zhang, Qian Cai, Yu Qian, Fengming Ran, Wuling Ou, Jun Wang, Qing Huang, Tianhua Ren, Guang Han, Feng Zhang, Wei Wei, Xinjun Liang, Huiting Xu, Sheng Wang, Lulu Shi, Shaozhong Wei, Sheng Hu

**Affiliations:** ^1^Department of Medical Oncology, Hubei Cancer Hospital, Tongji Medical College, Huazhong University of Science and Technology, Wuhan, China; ^2^Hubei Provincial Cancer Center, Wuhan, China; ^3^The Office of Hubei Provincial Cancer Prevention, Wuhan, China; ^4^The Cancer Quality Control Center of Hubei Province, Wuhan, China; ^5^Department of Radiological Intervention, Hubei Cancer Hospital, Tongji Medical College, Huazhong University of Science and Technology, Wuhan, China; ^6^Laboratory of Drug Discovery and Molecular Engineering, Department of Medicinal Plants, College of Plant Science and Technology, Huazhong Agricultural University, Wuhan, China; ^7^Department of Medicine, The University of Hong Kong-Shenzhen Hospital, Shenzhen, China; ^8^Department of Radiation Oncology, Hubei Cancer Hospital, Tongji Medical College, Huazhong University of Science and Technology, Wuhan, China; ^9^Department of Hepatobiliary and Pancreatic Surgery, Hubei Cancer Hospital, Tongji Medical College, Huazhong University of Science and Technology, Wuhan, China; ^10^The Administration of Cancer Clinical Trials and GCP, Hubei Cancer Hospital, Tongji Medical College, Huazhong University of Science and Technology, Wuhan, China; ^11^Radiotherapy Center, Hubei Cancer Hospital, Tongji Medical College, Huazhong University of Science and Technology, Wuhan, China; ^12^Department of Thoracic Surgery, Hubei Cancer Hospital, Tongji Medical College, Huazhong University of Science and Technology, Wuhan, China; ^13^Department of Gastrointestinal Surgery, Hubei Cancer Hospital, Tongji Medical College, Huazhong University of Science and Technology, Wuhan, China; ^14^College of Health Science, Huazhong Agricultural University, Wuhan, China

**Keywords:** coronavirus, COVID-19, SARS-Cov-2, cancer, treatment, immunotherapy, consensus

## Abstract

The sudden pandemic of SARS-Cov-2 (also known as novel coronavirus disease 2019, COVID-19) poses a severe threat to hundreds of millions of lives in the world. The complete cure of the virus largely relies on the immune system, which becomes particularly a challenge for the cancer subjects, whose immunity is generally compromised. However, in a constant evolving situation, the clinical data on the prevalence of SARS-Cov-2 for cancer patients is still limited. On top of a wide range of medical references and interim guidelines including CDC, NCI, ASCO, ESMO, NCCN, AACR, ESMO, and the National Health Commission of China, etc., we formed into a guideline based on our experience in our specialized cancer hospital in Wuhan, the originally endemic center of the virus. Furthermore, we formulated an expert consensus which was developed by all contributors from different disciplines after fully discussion based on our understanding and analysis of limited information of COVID-19. The consensus highlighted a multidisciplinary team diagnostic model with assessment of the balance between risks and benefits prior to treatment, individualizing satisfaction of patients’ medical needs, and acceptability in ethics and patients’ socio-economic conditions.

## Introduction

Oncologists are facing great challenges on treating cancer patients in the current pandemic of SARS-Cov-2 (also known as novel coronavirus disease 2019, COVID-19) (1). Given the lack of clinical guidance on cancer treatment in the set of the pandemic coronavirus, a rationale consensus for clinical cancer treatment becomes an urgent priority (2). Located in Wuhan, China, where the center of early COVID-19 breakout was, Hubei Cancer Hospital have tested 20,341 cases of SARS-Cov-2 nucleic acids for patients and caregivers, of which 30 were positive by June 24, 2020. At the meantime, the number of subjects took IgG and IgM tests of SARS-Cov-2 reached to 24,023 cases, of which 344 and 125 were showing positive, respectively. Although the recommendations for lung cancer was suggested based on ESMO management and treatment (3), general guidelines for cancer patients care and treatments are still in need because the unprecedented event not only directly claimed hundreds of millions of severe infection and lives, but also massively destructed the social structures and habits around the world. For the sake of cancer patient’s desperate expectation on the needs of personalized medical treatment and fully activation of the therapeutic potential of the multidisciplinary team (MDT) of the Hubei Cancer Hospital in Wuhan, we developed an expert consensus for treatment options on cancer patients during the pandemic of SARS-CoV-2 based on the coronaviruses pneumonia treatment guidelines posted by the National Health Commission (NHC) of China and the latest international research progresses from relevant literatures and professional organization websites including Center for Disease Control and Prevention (CDC), NCI, ASCO, ESMO, NCCN, and AACR. Three experts independently drafted the initial manuscript of the operating procedures, taking into account the actual coronavirus prevalent situation and medical capability of our hospital. After fully discussion and voting, we formulated the suggestions consented by all contributors from different disciplines.

## General Principles for Cancer Therapy

### Relationship Between Cancer Patients and New Coronary Pneumonia by COVID-19

So far, there were two Chinese studies of which 18 patients had cancer history in 1571 patients of COVID-19 and 12 patients suffered COVID-19 in 1571 cancer patients, suggesting the susceptibility to COVID-19 was higher than the overall population incidence, and concluding that cancer patients were most likely to be infected (4, 5). The Thoracic Cancers International COVID-19 Collaboration (TERAVOLT) registry indicated that as high as 76% patients with thoracic malignancies were hospitalized and 33% died of 200 patients with COVID-19 and thoracic cancers from eight countries, between March 26 and April 12, 2020 (6). Only 13 (10%) of 134 patients who met criteria for intensive care units (ICU) admission were admitted to ICU; the remaining 121 were hospitalized, but were not admitted to ICU (6). Patients with cancer have a higher risk to develop into serious consequences, which is defined as requiring invasive ventilation or ICU. Whether mortality could be reduced with treatment in intensive care remains to be determined. With improved cancer therapeutic options, access to intensive care should be discussed in a multidisciplinary setting based on cancer specific mortality and patients’ preference (6). However, it is not an ideal representative of the entire cancer subpopulation because of comparably small sample size (7). In addition, we did not notice a higher infection rate in cancer patients in our square cabin hospital, a specialized temporary hospital. Because the majority patients treated by square cabin hospital were in mild conditions, it may not represent common situation which is consistent with an epidemiological study covering cancer patients (8). Undoubtedly, patients in cancer treatment are at high risk, because they need to face two serious medical problems at the same time (9). TERAVOLT also indicated that 76% patients had non-small-cell lung cancer, and 74% were on therapy at the time of COVID-19 diagnosis, with 57% on first-line treatment (6). Therefore, it is essential to test SARS-CoV-2 in all lung cancer patients, where the early identification of SARS-CoV-2 may result in tailored management (10). In addition, a tiered approach based on the presumed risk of immunosuppression with different chemotherapy drug to testing could help provide patients with life-saving chemotherapy without jeopardizing their chances of benefit (11).

No evidence has been found that the infection rates are correlated with different histology such as breast or lung, treatment modality such as immunotherapy or tyrosine kinase inhibitor (TKI) treatment, or a subpopulation of patients such as children or elderly (12). As more evidence emerges, further information on COVID-19 from ASCO, CDC, ESMO, and NHC shall be continuously updated.

### Suggestions of Hygiene Instruction for Cancer Patients

It is necessary to spread strict hygiene instructions against COVID-19 to all the people, especially to vulnerable cancer patients. The cancer patients are required to make an appointment for COVID-19 screening after possible exposure or contact with patients verified with COVID-19 infection. Posters on general instructions of hygiene and symptoms of COVID-19 are suggested to exhibited in the entrance of hospitals or other eye-catching area for patients to understand and follow.

It is suggested to inform patients as much as possible to read and follow the existing comments and suggestions, such as manuals of new coronary pneumonia composed by us, as well as the New Coronavirus Pneumonia Prevention and Control Plan of the NHC (Sixth Edition), New Coronary Pneumonia Outpatient Rehabilitation Program of the General Office of the NHC (Temporary), New Coronavirus Pneumonia Diagnosis and Treatment Program (Seventh Edition), National Health Management Plan for New Crown Pneumonia Discharged Patients (Temporary), Psychological Counseling Work Program in New Coronary Pneumonia Outbreak and others by NHC (12–17).

### Management of Suspected Cases

**FIGURE 1:**
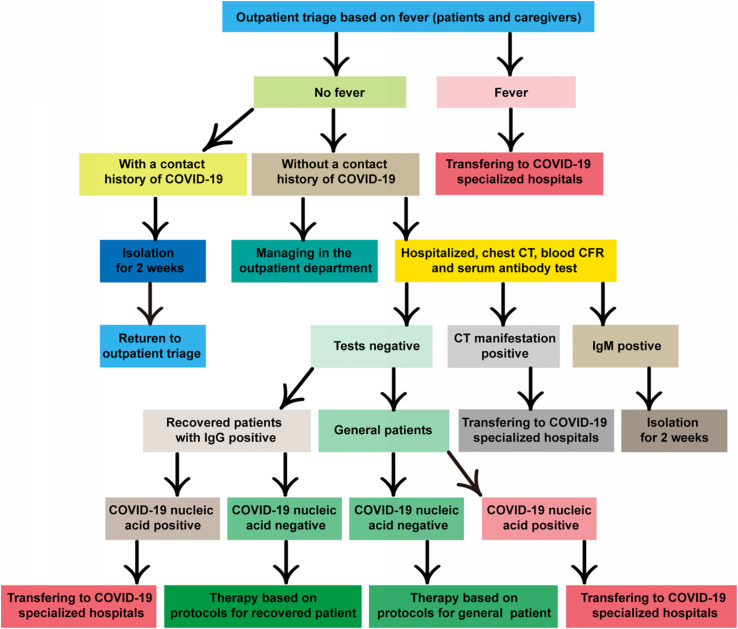
Flow diagram hospitalization during COVID-19 pandemic. The cancer hospital endeavors to take necessary measures to protect the right of an individual patient seeking medical assistance while keeps the institution efficiently running for protection of the medical professionals and many other hospitalized patients free of virus infection.

There is no specific guideline for the detection of COVID-19 (including nucleic acid, serum IgM/IgG antibodies, and chest CT) in cancer patients. For cancer patients with fever or other infection symptoms, relevant testing and isolation procedure should be conducted in accordance with NHC’s general medical and public health guidelines. The detailed and definite working protocols are available from New Coronary Pneumonia Screening Criteria for Cancer Patients in Hubei Cancer Hospital (Second Edition) or our hospital website (Figure 1). It shall be vigilant because the chance is increasing to meet asymptomatic infections in the later stage of the epidemic.

## General Principles for the Therapy of Common Patients With Cancer

### Surgery for Cancer Patients

According to the guidance from the CDC and the American College of Surgeons (ACS) (18), some general principles for all situations include: ➀ Based on medical urgency judgment and resource availability, patients should receive appropriately and timely surgical treatment before potentially infected patients are ruled out by COVID-19 test results. ➁ If it is clinically appropriate for patients, non-surgical treatment or rescheduling elective surgery should be considered. ➂ However, individual decision on the potential harm of delaying a cancer-related surgery should be made by clinicians. For example, patients with early hormone receptor-positive breast cancer may be suitable for months of endocrine therapy and a delayed surgery (19). ➃ In the perioperative later-period, patients might also try to recover at home, and keep communicating with MDT team at any time to reduce the chance of COVID-19 infection in hospital. ➄ Due to limited teamwork, emergency surgery at night is suggested to avoid. ➅ Aerosol generating procedure (AGP) increases the risk for medical staff, but it may not be avoided. Conditions likely causing AGP are tracheal intubation, bronchoscopy, chest tube, blood electrocautery, gastrointestinal tissues, body fluids, and laparoscopy/endoscopy. ➆ There is not enough data to recommend/oppose open or laparoscopy methods; however, the surgical team should choose the appropriate method to minimize the surgical time and to provide maximum safety for patients and healthcare providers. ➇ For the proposed surgery, the advantages of the MDT need to be fully adopted to reduce unplanned surgery (20).

### Anti-cancer Drug Therapy

At present, there is no direct evidence supporting change or discontinuation of chemotherapy, targeted therapy, or immunotherapy in cancer patients during COVID-19 pandemic (21). Therefore, routine suspension of key anticancer or immunosuppressive therapies is not recommended. Because it is difficult to balance the benefits of delayed or interrupted treatment in the course of the prevention of COVID-19, clinical decision should be individualized, such as the risk of cancer recurrence and the patient’s treatment tolerance (22). Here are the basic rules of decision making for cancer clinical physicians in the COVID-19 pandemic:

➀ For patients with significant remission of tumor undergoing maintenance therapy, it is considered to temporarily delay the chemotherapy. Chemotherapy of some patients can be transformed from intravenous to oral administration, but the medical team should be more vigilant to ensure that medicine is correctly administrated to patients.

➁ The decision of changing or interrupting chemotherapy should consider the urgency of treatment. For example, the ratio of risk to benefit of patients with untreated extensive small cell lung cancer is significantly different from patients with metastatic non-small cell lung cancer treated by pemetrexed maintenance. On the other hand, patients with aggressive hematological malignancies should be immediately treated to save lives.

➂ It is suggested to postpone adjuvant chemotherapy for 2 weeks if patients cannot arrive to the hospital for treatment, or it may also be delivered in another COVID-19 free medical center. Considering the convenience for patients and medical staff, chemotherapy in the outpatient department is feasible. If the benefits of adjuvant chemotherapy are likely to be less and there is a non-immunosuppressive option such as hormone therapy for ER + breast cancer, then the risk of COVID-19 needs to be preferentially considered.

➃ In high-risk and intensified chemotherapy regimens, the prophylactic growth factors and prophylactic antibiotics may have potential value for overall health improvement, thus these patients are relatively less vulnerable to COVID-19 complications.

➄ All of potential donors shall be tested for COVID-19 even in the absence of evidence of COVID-19 transmission through blood transfusion. Moreover, considering the insufficient supply, the blood products should be administrated only for urgent events.

➅ For patients having received long-term targeted treatment, oral drugs might be prescribed as long as possible such as for 1 to 2 months, and best obtaining from pharmacies but not hospitals.

➆ The affordability, i.e., economic toxicity should fully be considered while choosing treatment agents, because financial situation of each patient might have deteriorated significantly now and in future after the 2-month quarantine in pandemic.

➇ For patients of lung cancer, Neoadjuvant chemotherapies should be given top priority, as reported by NHS clinical guide for the management non-coronavirus, in patients with cancer requiring acute treatment. For patients with a new diagnosis of metastatic NSCLC, all standard options for first-line systemic therapy should be envisaged unaltered, including chemotherapy, immunotherapy, TKIs and different combinations, whereas delaying the treatment could compromise patient’s survival (high priority in ESMO) (3).

➈ For patients of metastatic NSCLC, immune checkpoint inhibitors (ICIs) schedule should be modified/delayed to reduce clinical visit, using 4-weekly nivolumab 480 mg or 6-weekly pembrolizumab 400 mg, instead of the standard 2-weekly, or 3-weekly (high priority). For patients on ICI for more than 12/18 months, delaying the subsequent cycle, omitting some cycles, or generally expanding intervals should be considered (3).

### Preventive Antiviral Therapy

Currently, there is no evidence or published study for preventive antiviral treatment of COVID-19 in immunosuppressed cancer patients. To date, potential antiviral drugs that have been used or clinically studied, such as oseltamivir, chloroquine, redecvir, lopinavir, and even lopinavir combined with ritonavir (23) have not been officially proven to inhibit COVID-19. Although there are more than 700 clinical trials ongoing in the world (24, 25), none agent has been applied in a preventive setting targeting cancer patients. We suggest that patients without digestive tract tumors or chemotherapy can receive prophylactic Chinese medicine against COVID-19 (26), because many drugs have some gastrointestinal side effects. As to SARS-CoV-2 vaccine, theoretically, it will be difficult to be applied clinically in the next 12–18 months, so it may not affect the first wave of the current pandemic. However, it can be very useful at a later time or in a post-pandemic population (27).

### Clinical Trials of Cancer Patients

According to the notification from the Hubei Provincial Drug Administration, currently it will not initiate new clinical trials that are not involved in new coronary pneumonia. New participants should be recruited for ongoing trials until the pandemic ending. The safety of trial participants and clinical investigators is paramount, which should be under vigorous personal protection (28). ASCO noted that clinical trials during the epidemic of COVID-19 are particularly challenging. Because of difficulties in meeting protocol-specified procedures including administering investigational product or adhering to protocol-mandated visits and laboratory/diagnostic testing (20). FDA has issued guidelines to provide general considerations on dealing with emerging issues (29). For patients already enrolled in ongoing clinical trials, the safety should always be a priority at all times through close communication among sponsors, investigators, research institution, GCP center, and independent ethics committee in accordance with GCP standards. Integrity of the clinical trials should be ensured in the greatest extent, with minimal deleterious effects to the health and benefits of the research participants (30). If participants may not be able to travel to the study site in a protocol-designated visit, sponsors and investigators should evaluate whether there are alternative security methods, such as telephone contact, virtual visits, alternative assessment sites, including local laboratories or imaging centers. The missing visits of a study should be recorded. During clinical research visits, even if a COVID-19 screening procedure is mandatory before receiving research productions, it is no required to report amendments to the protocol unless the sponsor will incorporate the collected data into new research objectives. Intravenous injection should be decentralized into a longer range of time because of relatively limited capacity compared with non-epidemic. The additional costs in travel and screening should be reimbursed through the sponsor as much as possible.

An additional safety monitoring especially for the infection, is recommended to all participants, even if they no longer use research products, such as withdrawal from study treatment (29). For those who have been infected by COVID-19, the clinical study should be suspended because direct and indirect influence of virus is unclear, although a case of male lung cancer receiving investigational anti-PD-1 antibody in our center, infected by COVID-19 in community, has similar recovery process about 3 weeks to others. When the quarantine expires, infected participants should be isolated for 8 weeks to evaluate potential adverse effects for a decision of the next interventions strategy in accordance with NHC.

### Other Treatment Options

At present, there is not specific suggestion on any other kinds of treatment for cancer patients, such as radiotherapy or ablation treatments. Overall, intensifying palliative care for cancer patients, and good management of common symptoms based on different grades, such as pain, dyspnea, nausea, vomiting, and fatigue can improve the quality of patients’ life, even enhancing survival rate (31, 32). Psychological counseling service for patients and their families should be provided by psychotherapists and social workers, if necessary, transferring is necessary to a psychiatrist for further intervention. It is recommended to temporarily defer cancer screening procedures, such as screening mammograms and colonoscopy (16, 21). Of course we can observe several cases of the chest and mammary glands tumors during the screening for COVID-19 pneumonia by CT scan.

## Managements of Cancer Patients Recovering From COVID-19 Infection

### Definition of Patients Recovering From COVID-19 Infection (Pneumonia Only)

The definition of recovered cancer patients is, who is healed from new coronary pneumonia contracted with COVID-19, meeting the discharge standards of the New Coronavirus Pneumonia Diagnosis and Treatment Program (Seventh Edition; National Health Commission, China) (12). It should be strictly assessed of clinical symptoms, signs, laboratory, and imaging, as well as detailed diagnosis and treatment summary from designated hospitals. A clear follow-up records of 3–4 weeks is expected after the 2 weeks isolation.

### General Requirements for Anti-tumor Therapy for Cancer Patients Recovering From New Coronary Pneumonia

Subject who is appropriately considered to cancer treatment has to have an interval time over 2 weeks after quarantine isolation plus twice negative nucleic acid tests with PS score < 3.

The exclusion criteria follow as: ➀ Resting heart rate > 100 beats/min. Blood pressure is also an exclusion factor with threshold of <90/60 mmHg, >140/90 mmHg, or blood pressure fluctuations exceeding the baseline by 20 mmHg. Significant discomfort such as dizziness and headache and the blood oxygen saturation ≤ 95% are also exclusion indicators. ➁ Subjects are to be excluded with symptoms of cough, expectoration, fever and diarrhea. Further exclusion symptoms include serious dyspnea due to impaired lung function or respiratory muscle weakness with shortness of breath after exercise, physical dysfunction manifested by obvious weakness and muscle aches, mental dysfunction, including anger, anxiety, and depression.

The treatment might include patient with a history of severe or critical type of pneumonia due to conventional comorbidities such as diabetes, hypertension, fractures, or skin inflammation or allergies, but not due to impaired lung function caused by viral infection.

### Therapeutic Recommendations and Options

It is recommended to evaluate the risk or benefit for any patient who is recovered from SARS-CoV-2 infection, because the new coronary virus is complicated by many uncertain factors (33). For example, the CDC suggested to closely monitor patients with immune incompetence (34), e.g., administration of immunosuppressive reagents, bone marrow or solid organ transplantation, genetic immune deficiency, poor control for human immunodeficiency virus, etc., because they might reappear to positive nucleic acid tests and most probably continue to shed SARS-CoV-2 virus even after their recovery from a specific infection (9). In addition, SARS-CoV-2 virus enters into the human respiratory epithelial cells dominantly via viral spike protein binding to angiotensin-converting enzyme 2 (ACE2) receptor expressed on cell surface (35). Besides strong expression in type II alveolar cells, ACE2 is also highly expressed in heart tissue, physiopathologically down regulating of the abnormal activation of renin-angiotensin system in the setting of hypertension, congestive heart failure and atherosclerosis. Thus, COVID-19 infection correlates with an increase of incidence and mortality of cardiovascular diseases and it poses an impact on clinical cancer care (36).

#### Surgery

When surgery is performed for patients recovered from SARS-CoV-2 infection, surgery team should execute completely personal protective equipment, including N95 mask, and powered air-purifying respirator that is specially designed for operation room (37, 38). For patients with thoracic tumor, the timing and type of surgery are principally determined by the severity degree of disease, for example, these conditions can be rescheduled to delay 1–2 months, including ground glass nodules with <50% solid or tumors, solid nodules or lesions <2 cm in greatest dimension, indolent behavior such as carcinoid or slow growth pattern, and asymptomatic and non-bulky thymoma (32). An alternative surgery may be an optimal choice in some cases, for example, superficial esophageal cancer (T1a/b) can be surgically removed by endoscopy. When certain patients are potentially suitable for adjuvant therapy, administration of neoadjuvant therapy should be adopted, e.g., neoadjuvant chemotherapy or stereotactic ablation radiotherapy is feasible to treat patients with lung cancer and lesion diameter equal or less than 5 cm (20, 38).

In more advanced cases, the surgery should be considered to carry out if the survival time of patients is compromised in the next 2–3 months. These include: patients had solid or mainly solid (>50%) of lung cancer with lesions >2 cm in greatest dimension and clinically negative lymph node, or with positive lymph node, esophageal cancer with ≥T1b, no alternative regimen for chest wall tumor with highly potential malignancy, or obtaining stage treatment including mediastinum macroscopy examination, diagnostic VATS for pleural disseminated lesions, or symptomatic mediastinal tumor but not suitable for diagnostic biopsy (39). Robot assisted surgery may reduce not only hospital stay for patients that urgently need complex-oncological-surgery, but also the number of directly exposed medical staff in comparison to open or conventional laparoscopic surgery under COVID-19 circumstances (40).

Surgery should be performed as soon as possible when potentially emergent situation is expected to become progressive in the next few days, including esophageal cancer with perforation but not-sepsis, cancer related infection critical but not septicemia, e.g., cytoreduction for intestinal obstruction or pneumonia caused by tumor, and surgical complications including hemothorax, empyema and titanium plate repairing for infectious disease in patients with stable hemodynamics (38, 41).

Finally, if there are no other limitations, an emergency surgery shall be carried out in the next few hours for esophageal cancer with perforation and septicemia, airway obstruction, cancer related septicemia or heavy bleeding, surgical complications such as active bleeding without effective conservative therapy, airway rupture, anastomotic leakage with sepsis. For patients with other tumor rather than thoracic tumor, the indication of surgery might refer to other patients with non-infection (38, 39, 41, 42).

#### Chemotherapy

Though it is a difficult option to delay or proceed chemotherapy for the cancer patients who had recovered from COVID-19 infection, we still recommend that adjuvant chemotherapy for potential cure may continue to be administered with a regular dosage. Postponing chemotherapy may lead to disease deterioration and losing opportunity to slow down the cancer development of patients with metastatic diseases due to a missing therapeutic window period (43). However, high-dosage chemotherapy is generally forbidden in this setting, as whether host immune system previously attacked by SARS-CoV-2 can withstand the toxicity from chemotherapy is still a black box. It has been reported that COVID-19 potentially cause damage to many organs in addition to lung, including but not limited to heart, liver, kidney, brain, and even eyes, and cause a variety of complications (44–52) (Table 1). Furthermore, whether the reserved function of vital organs can be restored immediately or not remain elusive. Therefore, the functionality of main organs should be carefully assessed when patients begin to take initial chemotherapy.

**TABLE 1:** The relationship between complications and outcome of patients with COVID-19.

	Complications	Results		
		All(*N* = 102)	Non-survivors(*N* = 17)	Survivors(*N* = 85)
Cao et al. (46)*	Shock	10(9.8)	7(41.1)	3(3.5)
	ARDS	20(19.6)	15(88.2)	5(5.9)
	Acute infection	17(16.7)	14(82.4)	3(3.5)
	Acute cardiac injury	15(14.7)	12(70.6)	3(3.5)
	Arrhythmia	18(17.6)	12(70.6)	6(7.1)
	Acute kidney injury	20(19.6)	15(88.2)	5(5.9)
	Acute liver injury	34(33.3)	13(76.5)	21(24.7)
	Lymphopenia	78(76.5)	17(100.0)	61(71.8)
Wang et al. (47)*		All(*N* = 138)	Non-ICU(*N* = 102)	ICU(*N* = 36)
	Shock	12(8.7)	11(30.6)	1(1.0)
	Acute cardiac injury	10(7.2)	8(22.2)	2(2.0)
	Arrhythmia	23(16.7)	16(44.4)	7(6.9)
	ARDS	27(19.6)	22(61.1)	5(4.9)
	AKI	5(3.6)	3(8.3)	2(2.0)
		All(*N* = 1099)	Non-severe(*N* = 926)	Severe(*N* = 173)
Guan et al. (48)*	Septic shock	12(1.1)	1(0.1)	11(6.4)
	Acute respiratory distress syndrome	37(3.4)	10(1.1)	27(15.6)
	Acute kidney injury	6(0.5)	1(0.1)	5(2.9)
	Disseminated intravascular coagulation	1(0.1)	0	1(0.6)
	Rhabdomyolysis	2(0.2)	2(0.2)	0
	Physician-diagnosed pneumonia	972/1067(91.1)	800/894(89.5)	172/173(99.4)
Zhou et al. (49)*		All(*N* = 191)	Non-survivors(*N* = 54)	Survivors(*N* = 137)
	Sepsis	112(59%)	54(100%)	58(42%)
	Respiratory failure	103(54%)	53(98%)	50(36%)
	ARDS	59(31%)	50(93%)	9(7%)
	Heart failure	44(23%)	28(52%)	16(12%)
	Septic shock	38(20%)	38(70%)	0
	Coagulopathy	37(19%)	27(50%)	10(7%)
	Acute cardiac injury	33(17%)	32(59%)	1(1%)
	Acute kidney injury	28(15%)	27(50%)	1(1%)
	Secondary infection	28(15%)	27(50%)	1(1%)
	Hypoproteinemia	22(12%)	20(37%)	2(1%)
	Acidosis	17(9%)	16(30%)	1(1%)
Huang et al. (50)*		All(*N* = 41)	ICU(*n* = 13)	No ICU care(*n* = 28)
	Acute respiratory distress syndrome	12(29%)	11(85%)	1(4%)
	RNAaemia	6(15%)	2(15%)	4(14%)
	Cycle threshold of RNAaemia	35.1(34.7–35.1)	35.3(35.1–35.1)	34.8(34.1–35.4)
	Acute cardiac injury	5(12%)	4(31%)	1(4%)
	Acute kidney injury	3(7%)	3(23%)	0
	Secondary infection	4(10%)	4(31%)	0
	Shock	3(7%)	3(23%)	0
Chen et al. (51)*		All(*N* = 33/99)		
	ARDS	17(17%)		
	Acute renal injury	3(3%)		
	Acute respiratory injury	8(8%)		
	Septic shock	4(4%)		
	Ventilator-associated pneumonia	1(1%)		

*Reported complications and outcome of patients with COVID-19 from some important studies.

Being a homolog of ACE, ACE2 [SARS-CoV-2 enters into cellular membrane depended on ACE2 and TMPRSS2 (53, 54)] transforms angiotensin II into angiotensin 1–7, and subsequently antagonizes the vasoconstriction that is physiologically regulated by the renin-angiotensin system (55). It is disputed on whether ACE inhibitor (ACEI) and angiotensin receptor blocker (ARB) increase the level of ACE2 or not. Nevertheless, lung injury was successfully alleviated by both recombinant ACE2 and losartan in mouse models (56). Nearly all academic organizations claim that ACEI, ARB, or other RAAS antagonists should not be changed without other purpose, because no evidences available in term of the benefit and risk of these agents (57).

Some drugs that have showed potential cardiac side effects, such as anthracycline, paclitaxel, herceptin, etc., should be withdrawn or postponed in patients with insufficient cardiac function induced by viral myocardial injury (58). Meanwhile, ACE2 showed positive expression in intestinal epithelium cells, vascular endothelium cells and kidney, so SARS-CoV-2 infection can possibly lead to multiple organ dysfunction. For safety reasons, we suggest that platinum should be avoided for patients previously experienced renal dysfunction. More importantly, in some cases, irinotecan, and fluorouracil are considered not safe if patients have suffered from diarrhea because of infection. Collectively, risk-benefit assessment should come first before medical interfering and other details may refer section “Anti-cancer drug therapy.”

#### Targeted Therapy

A variety of targeted drugs with distinct molecular targets such as TKIs, have been made commercially available. The distinction of adverse reaction may not only depend on the type of reagents/drugs, but the heterogeneity of patient population (59). We are particularly concerned about cardiovascular, pulmonary and gastrointestinal side effects, as the toxicity profile is clearly overlapped with viral pneumonia (44, 47, 52, 60).

##### Adverse effects of cardiovascular system

Cardiovascular adverse effects are usually reversible after discontinuation, but not always. These adverse effects include left ventricle (LV) dysfunction with or without symptoms, conduction abnormalities/arrhythmias, arterial hypertension, and thromboembolism. It is important to understand the side effects of these drugs (61).

###### LV dysfunction

The most common drugs linked to this side effect are HER-2 inhibitors, such as trastuzumab and patozumab. It has been proved that combination of these two drugs does not result in a significant increase in cardiac side effects. Lapatinib is an orally dual EGFR/HER2 TKI that induce both asymptomatic and symptomatic heart events. Other kinase inhibitors are also responsible for myocardial injury, e.g., ALK, FGFR4, MEK1, and MEK2 (62).

###### Hypertension

Hypertension is a classic on-target side effect related to inhibitors involving VEGF pathway, such as bevacizumab, aflibercept, and VEGFR multikinase inhibitors including sorafenib, sunitinib, regofinib, apatinib, and arotinib. Although SARS-CoV-2 binds to ACE2, no evidence indicates that it is necessary to replace drug against hypertension during the epidemic period of COVID-19. In contrast, postural hypotension resulting from thalidomide and bortezomib is a manifestation of autonomic nervous dysfunction and part of drug-induced neurological diseases (59, 61, 62).

###### Abnormal conduction

Sinus bradycardia is a known adverse effect of thalidomide. Moreover, 19% of patients receiving crizotinib suffered from sinus bradycardia. Other kinase inhibitors, such as sunitinib, sorafenib, pazopanib, vandetanib, nilotinib, dasatinib, vemurafenib, and histone deacetylase inhibitors including romidepsin, panobinostat, show an abnormal cardiac conduction too (60, 62, 63).

###### Bleeding and thromboembolism events

The increasing risk of arterial ischemia/thromboembolism (ATE) rather than venous thromboembolism (VTE) correlated with VEGF pathway inhibitors, such as sorafenib, sunitinib as well as the combination of bevacizumab and chemotherapy. Thalidomide and its analog exhibited significant increase in the risk of VTE with combination to other drugs such as dexamethasone or adriamycin (64).

##### Adverse effects of pulmonary

The incidence of drug-induced non-infective pneumonia/interstitial lung disease (ILD), which particularly intimidated to us, including asymptomatic X-ray manifestation, non-specific inflammatory infiltration and even fatal cases, was 0.1–15% with malignant disease after administration of novel antitumor targeted drugs (59, 65). Lung toxicity is particularly common for mTOR inhibitors (66). In most cases, cough and dyspnea with or without fever are frequent symptoms. In general, pneumonia occurs at the first 6 months of treatment and can be detected as early as 2 months after treatment by X-ray examination. CT scanning presents distinct manifestations from the most common diffuse patchy ground glass lesion to traditional drug-induced ILD, similar to acute interstitial pneumonia, and acute respiratory distress syndrome. It has been reported that pan-PI3K inhibitors, EGFR inhibitors or monoclonal antibody drugs were closely linked to ILD. Bortezomib, thalidomide and its analog, imatinib, MET inhibitors including crizotinib and tivantinib, HSP inhibitors (e.g., 17DMAG) were reported to show drug-induced ILD, too. Pleural effusion occurred in 16% to 54% of patients treated with dasatinib, and another rare toxicity for BCR/ABL inhibitors was pulmonary arterial hypertension (60, 67).

##### Adverse effects of gastrointestinal/hepatobiliary

Diarrhea related inhibition of Notch signaling can be triggered by application of EGFR/Raf/MEK pathway inhibitors, VEGFR, and ABL multikinase inhibitors, and PI3K/Akt/mTOR pathway inhibitors. Treatment should be carried out immediately to avoid life-threatening dehydration resulted from further uncontrolled diarrhea. The treatment includes active administration of antidiarrheal/anti-peristaltic agents such as loperamide or diphenoxylate and atropine. Isolated hyperbilirubinemia, which depends on the unconjugated proportion, can be a potential side effect of small molecular TKIs such as erlotinib, sorafenib, regofinib, pazopanib, and nilotinib. Therefore, it is especially crucial to strict monitoring during the whole patient care (60, 68, 69).

Consequently, we suggest targeted therapy might be regularly used for rehabilitation patient. However, the rational arrangement of anticancer targeted drugs is extremely important, as the predominant trepidation is potential incomplete restoration or/and long-term influence from immune memory (70). Anyway, toxicities of vital organs especially pulmonary have to be closely monitored, although the incidence is not common (details in section “Monitoring of adverse effects”).

#### Immunotherapy

The wide range of immune-related adverse effects associated with immunotherapy which induces an unexpected activation of cytotoxic T cells may injure almost all of organs. Besides, impact of immune-related adverse events (irAEs) show notable disparity among different agents, regimens and schedule. Toxic effects seem to be more serious in anti–CTLA-4 than that of anti- PD-1/PD-L1 monotherapy, while multiple concurrent irAEs are more common in combination therapy than in monotherapy. Accurately, single ipilimumab caused colitis/diarrhea in predominant cases (70%), but hepatitis (16%), and pneumonitis (8%) in smaller proportions. Anti–PD-1/PD-L1 monotherapy, by contrast, has a wide distribution of fatal irAEs including pneumonitis (35%), hepatitis (22%), colitis (17%), neurologic events (15%), and myocarditis (8%). Mortality in combination therapy are most often owing to colitis (37%), myocarditis (25%), hepatitis (22%), pneumonitis (14%), and myositis (13%). Myositis and myocarditis frequently co-occur (71–73).

Due to lack of prospective trials targeting irAEs treatment, clinical information is only obtained from small series of studies, case reports and expert opinions. Current guidelines recommend a step-by-step approach, starting with high-dose steroids in the first instance and then increasing immunosuppressive agents as needed (74). However, rapid immunosuppression is a prioritized option because it is presumed with immediate efficacy for ICI-related myocarditis with fulminant clinical presentation, and the higher associated morbidity and mortality rate. Biomarker-based approaches to dose immunosuppressants are under study and will probably help make therapeutic decisions. For patients with predominant T-cell infiltrate, the preferably approach is anti-IL-6, and if it is not available, anti-IL-1 receptor, anti-IL-12, and anti-IL-23 blockade are also acceptable (75).

In case of B-cell and plasma cell infiltration, anti-B-cell might be an optimal strategy such as blockade by anti-CD20 and anti-B-cell activating antibodies (76). When infiltrate is dominated by neutrophilic and monocytic with or without granulomas, targeted anti-TNFα strategy is the best option. Calcineurin inhibitors and mycophenolate mofetil have significant inhibitory effects on T cell responses. However, it should be avoided in patients with immunogenic tumors, especially in the hope of likely full recovery (77).

The inflammatory response to SARS-CoV-2 infection is thought to underpin COVID-19 pathogenesis through inflammatory protein expression and different T cell subpopulation (78). Therefore, it should be pointed out that immune insufficiency or misdirection may increase viral replication and make tissue damage. In contrast, overactive immune responses may induce immunopathological conditions (79). Which one in such dichotomous mechanisms is important to COVID-19 pathogenesis, remains unknown. All clinical immunological studies thus far have analyzed blood samples collected over a range only from the period of illness onset to rehabilitation. In severe cases, a contradiction phenomenon appears because it exists both immune function impairment and over activation, which was shown in a study. Of 452 patients with COVID-19 recruited in Wuhan, 286 were diagnosed as severe infection, who tended to have lower counts of lymphocytes, higher leukocytes and neutrophil-lymphocyte-ratio. In most of severe cases, infection-related biomarkers and inflammatory cytokines elevated including TNF-α,IL-2R, and IL-6 (80). On the other hand, the number of T cells significantly decreased including helper T cells, T_*reg*_ cell and memory T-cell, and it is more obvious in severe cases with COVID-19.

**FIGURE 2:**
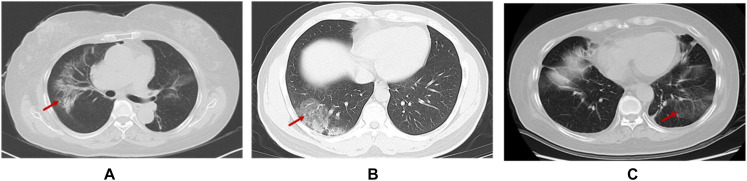
CT scan of patients contracted with ICI-pneumonitis **(A)** and viral pneumonitis [**(B)** (90) and **(C)**], respectively. **(A)** Female, 71 years old, advanced lung adenocarcinoma receiving immunotherapy for 8 months in our cancer hospital **(C)** Female, 62 years old, advanced lung adenocarcinoma, receiving chemotherapy in our cancer hospital.

Finally, not every patient can generate antibodies or sufficiently effective antibodies against COVID-19 (81). Moreover, the data shown antibody against MERS-CoV could not protect infection with a pseudovirus bearing bat MERSr-CoV (82). Although the possibility of reactivation of the virus is rare in the recovered patients (83), the specific time window and mechanism of recurrence are also unclear still now. Consequently, we are perspicaciously concerned whether the virus can integrate into the host genome like other virus, such as HBV (Hepatitis B virus), EBV (Epstein-Barr virus), HSV (Herpes simplex virus), and HPV (Human papillomavirus), which chronically remain in the host even without symptoms, as no study to confirm until now. Moreover, increasing evidence shows that SARS-CoV2 has neuroinvasive potential apart from the respiratory tract damage (84) like HSV neurotrophic characteristic. Most importantly, the pneumonia induced by immunotherapy is highly prevalent (10–19%) (85, 86). Moreover, its CT manifestations characterized by peripheral distribution, traction bronchiectasis, reticular opacities, ground glass opacities, centrilobular nodularity, and honeycombing, are highly similar to viral pneumonia, which are difficult to distinguish in general clinic especially for those with asymptomatic infection (Figure 2) (87–89).

**FIGURE 3:**
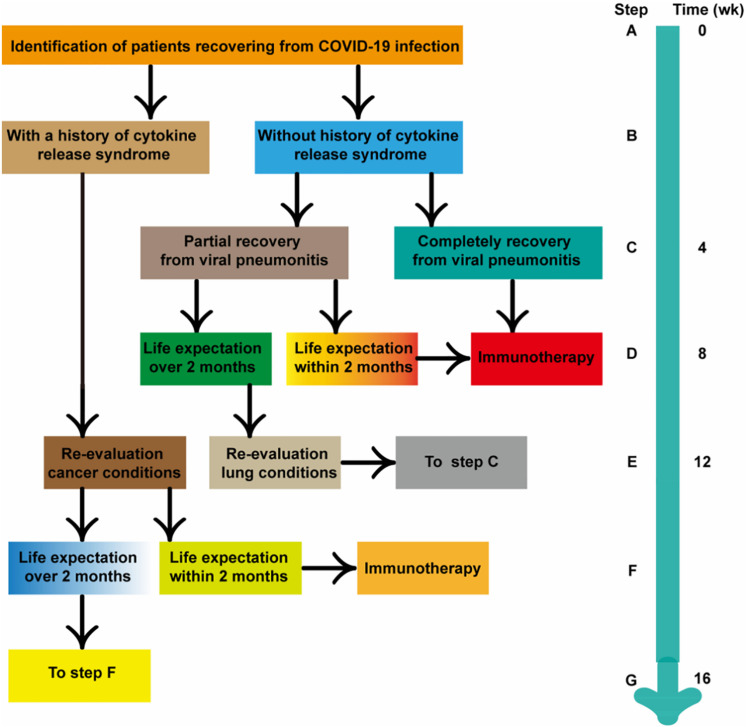
Conceptual flow diagram for prioritizing systemic immunotherapy for cancer patients recovered from COVID-19 infection.

It is reported that anti-tumor treatment including chemotherapy and immunotherapy could be performed after complete recovery and a 2-weeks medical observation for confirmed or suspected coronavirus patient (91). However, we suggest to suspend systemic immunotherapy during the 8 weeks of immediate recovery period is a suitable choice (details in Figure 3) (92). But, any decisions to postpone, discontinue or modification of the immunotherapy should be individualized in accordance with the overall assessments of treatment benefits exceeding the risks of cancer progression and side effect. There was one patient of lung cancer recovering from COVID-19 infection (pneumonia only) in our hospital, who had received ICI for more than 12 months before the infection. According to our recommendation, continued immunotherapy was given after 8 weeks of follow-up, while his IgG antibody was still positive. No side effects of immunotherapy appear and the novel coronavirus pneumonia don’t not relapse at present.

#### Combined Therapy

In our opinion, the chemotherapy can be combined with targeted therapy after evaluation of risk and benefit, as long as toxicity spectrum composed of different drugs did not overlap and dodge the disorders of SARS-CoV2. A paradigm is that anthracycline plus HER-2 antibody or platinum plus anti VEGFR agents should be separately applied for heart or kidney damage by COVID-19. The combination of two targeted drugs such as rituximab combing lenalidomide is not recommended to take in the 8 weeks of immediate recovery period, as it is difficult to predict the side effects in special subpopulation, although their combination is safe in general cancer patients. Typical side effects form traditional Chinese medicine should also consider. Similarly, it is also recommended not to select combination strategy containing systematic immunotherapy, including immune-checkpoint inhibitors, CAR-T cell, and cytokine therapy.

#### Endocrine Therapy

The side effects of endocrine therapy in cancer treatment are usually mild, however, 65% patients treated with palbociclib showed a grade of 3–4 neutropenia, of which, 34% patients need less dosage. While 4% patients who received palbociclib combined with fulvestrant discontinued the treatment due to adverse events (93, 94). Therefore, it is very important to closely monitor adverse events.

#### Radiation Therapy

Radiation therapy for thoracic tumor is a great challenge to radiation oncologists who should inform the possibility of harm or injury to patients. Radiotherapy kills cancer cells, but it also leads to obvious lung fibrosis in radiation field and triggers the release of inflammatory factors. Other adverse effects include increasing the infiltration of immune cells to tumor, reshaping the tumor immune microenvironment and motivating abscopal effect (95, 96). Based on the CT data of 90 non-cancer patients, after a median of 16 days of treatment, only 4 of the 70 patients discharged from the hospital showed complete disappearance of lung abnormalities on the last CT scan, which was 2 days before discharge. Residual lesions were present in the other 66 patients, mainly showing abnormally opaque ground glass, or irregular lines and interfaces (97).

Therefore, firstly, we suggest hypofractionated radiotherapy may be more appropriate for the sake of time saving and normal tissue protection, such as left-sided breast RT using 28.5–6 Gy in 5 fractions with deep inspiration breath hold over 1–2 weeks might also be considered for selected patients (98, 99). However, conventionally fractionated daily radiotherapy has been also shown to increase the local expansion of pre-existing T-cell clones and diversification of the T-cell receptor repertoire, so the optimal radiotherapy dose and fractionation to invigorate the immune response remains unknown (100), especially for thoracic cancer patients with lung disorders of SARS-CoV2.

Secondly, if possible, palliative care with similar effects to radiotherapy should be in the top priority but not radiation therapy. Thirdly, the interval time from complete resolution of pulmonary inflammation should be at least 4 weeks for thoracic rather other anatomic sites. Fourthly, for thoracic irradiation, all image data must be examined during pneumonia period for better designing a detailed radiotherapy plan. Planned Radiation fields involving heart should be established based on the cardiac function during the infection and recovery period. At last, for patients without emergency situations, i.e., adjuvant radiotherapy for breast cancer patients, radical radiotherapy for low-moderate risk prostate cancer, it is suggested to postpone the radiotherapy (21, 98). In addition, we suggest that special radiotherapy room and equipment for these patients should be arranged to eliminate the anxious sentiment of the general cancer patients.

### Monitoring of Adverse Effects

#### Monitoring of the Novel Coronavirus Pneumonia

**FIGURE 4:**
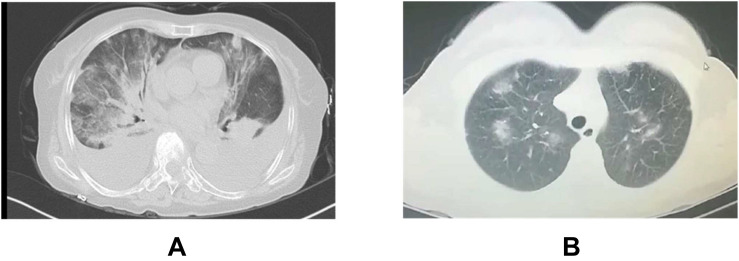
Typical CT of TKIs-pneumonitis **(A)** and viral pneumonitis **(B)** A: Female, 65 years old, advanced lung adenocarcinoma, TKI after 3 months in our cancer hospital B: Female, 42 years old, outpatient CT scanning for cough in our cancer hospital.

It is indispensable to differentiate the pneumonia from viral infection and drug side effects. Firstly, SARS-CoV-2 nucleic acid, and serum antibody detection should be tested every week for patients in rehabilitation. Secondly, the chest CT should be examined every month. Fortunately, we found TKIs-induced pneumonia with bilateral distribution of diffuse ground-glass opacities (101) is easier to distinguish from SARS-CoV-2 pneumonia (Figure 4), compared to ICIs-induced pneumonia, however, the differential diagnosis for atypical cases are still difficult which requires the assistance from MDT team. Thirdly, we should also be alert to the contingency that organic injuries from viral recurrence and anti-tumor treatment occur simultaneously. If there is recurrence suspect of the novel coronavirus pneumonia, it has to be immediately reported to the medical administration department according to the relevant administration protocol (9, 12). Patients in clinical trials are recommended to systematically review under GCP guidance.

#### Monitoring of Adverse Effects of Antitumor Treatment

Routine blood cell analysis and biochemical tests (at least including enzymes indicated myocardial injury) are performed once a week in the first 4 weeks and then every other week for the next 4 weeks. These tests are highly recommended for patients who have a history of immunotherapy or myocardial injury. Some other tests, such as ECG, urination analysis, infectious causes, procalcitonin, tumor markers, cardiac function assessment, and chest CT, are performed as needed.

## Ethical Considerations

It is necessary to sign informed consent for all patients. Moreover, for patients recovered from COVID-19 infection, special document named as *informed consent of anti-tumor therapy for the cancer patient recovered from novel coronavirus pneumonia* which comprehensively states the probability of previously unforeseen harm and financial lose, should also be signed. Patients are advised to consult their families or guardians, as well as lawyers, about the risks and benefits of the treatment. We oppose repeatedly and inappropriate screening for COVID-19 infection due to excessive stress and apprehension, which lead to therapy discontinuing and cost increasing, especially for patients with poor economic or carcinoma conditions.

The prognoses are often poor for advanced cancer patients even assisted with mechanical ventilation. Clinicians should discuss hospice and palliative care with patients and their families. During the period of novel coronavirus pneumonia outbreak, medical equipment is in severe shortage. Due to the limited medical resources, oncologists must also carefully consider the most effective treatment and minimal cost to the patients who may benefit the most to relieve symptoms or save lives (102).

In the end, the risk of viral infection should be minimized and the benefit of anti-tumor should be maximized through planned surgery, reasonable chemotherapy, targeted therapy, radiotherapy and immunotherapy for cancer patient during COVID-19 outbreak. Each decision for patient has to be based on individualized risk/benefit assessment under a graded-framework for prioritizing cancer care (20, 103–105). This precautionary principle in decision making will help cancer patients get through the viral pandemic smoothly and safely.

## Data Availability Statement

The datasets presented in this study can be found in online repositories. The names of the repository/repositories and accession number(s) can be found in the article/supplementary material.

## Author Contributions

SD, CL, XH, SWe, and SH developed the first outline, based on the medical references and interim guidelines including CDC, NCI, ASCO, ESMO, NCCN, AACR, ESMO, and the National Health Commission of China and recommendations from different disciplines after full discussion of all authors. SH, JZ, QC, YQ, FR, JW, QH, XL, and HX were responsible for the patients’ management in Department of Medical Oncology and discussed the treatment recommendations. SWe, FZ, and SWa were responsible for the patients’ management in Department of Surgery and discussed the treatment recommendations. GH and WW were responsible for the patients’ management in Department of Radiation Oncology and discussed the treatment recommendations. XH and TR were responsible for epidemiology consultant of COVID-19. LS was responsible for the clinical trials management of cancer patients. SD, CL, XH, SWe, and SH coordinated the authors’ contributions and engaged for competencies and expertise in the management of cancer patients. All the authors were involved in writing and when the first draft had been developed, all the authors refined the contents with feedbacks, and comments, incorporated by SD, CL, XH, SWe, and SH. All the authors approved the final draft before the submission.

## Disclaimer

Recommendations herein are not the mandatory standard care as the information is not comprehensive and continually updated for cancer patients during SARS-CoV-2 pandemic. Therefore, clinicians should carry out clinical practice to treat individual patients based on availability of medical resources. In addition, this guideline assumes no responsibility for any injury to relevant personnel or damage of property due to following of this information. The strategies outlined in this consensus are not intended to substitute the independent professional judgment as unique understanding of COVID-19 risk in different countries, hospitals, and healthcare environments in the pandemic stage.

## Conflict of Interest

The authors declare that the research was conducted in the absence of any commercial or financial relationships that could be construed as a potential conflict of interest.

## References

[1] Wu Z McGoogan JM. Characteristics of and important lessons from the Coronavirus disease 2019 (COVID-19) outbreak in China: summary of a report of 72 314 cases from the Chinese center for disease control and prevention. *JAMA.* (2020). 10.1001/jama.2020.2648 [Epub ahead of print], 32091533

[2] Wang H Zhang L. Risk of COVID-19 for patients with cancer. *Lancet Oncol*. (2020) 21:e181 10.1016/S1470-2045(20)30149-2 PMC7129735 32142621

[3] Passaro A Addeo A Von Garnier C Blackhall F Planchard D Felip E ESMO management and treatment adapted recommendations in the COVID-19 era: lung cancer. *ESMO Open.* (2020) 5 (Suppl. 3):e000820. 10.1136/esmoopen-2020-000820 32581069 PMC7319703

[4] Liang W Guan W Chen R Wang W Li J Xu K Cancer patients in SARS-CoV-2 infection: a nationwide analysis in China. *Lancet Oncol.* (2020) 21:335–7. 32066541 10.1016/S1470-2045(20)30096-6 PMC7159000

[5] Yu J Ouyang W Chua MLK Xie C. SARS-CoV-2 transmission in patients with cancer at a tertiary care hospital in Wuhan, China. *JAMA Oncol.* (2020) 6:1108–10. 32211820 10.1001/jamaoncol.2020.0980 PMC7097836

[6] Garassino MC Whisenant JG Huang LC Trama A Torri V Agustoni F COVID-19 in patients with thoracic malignancies (TERAVOLT): first results of an international, registry-based, cohort study. *Lancet Oncol.* (2020) 21:914–22. 32539942 10.1016/S1470-2045(20)30314-4 PMC7292610

[7] Xia Y Jin R Zhao J Li W Shen H. Risk of COVID-19 for patients with cancer. *Lancet Oncol.* (2020) 21:e180 10.1016/S1470-2045(20)30150-9 PMC7130057 32142622

[8] D’Antiga L. Coronaviruses and immunosuppressed patients: the facts during the third epidemic. *Liver Transpl.* (2020) 26:832–4. 32196933 10.1002/lt.25756

[9] Ja‘zieh AR Alenazi TH Alhejazi A Safi F Olayan A. Outcome of oncology patients infected with Coronavirus. *JCO Glob Oncol.* (2020) 6:471–5. 32196389 10.1200/GO.20.00064 PMC7124938

[10] Passaro A Peters S Mok TSK Attili I Mitsudomi T de Marinis F. Testing for COVID-19 in lung cancer patients. *Ann Oncol.* (2020) 31:832–4. 32278879 10.1016/j.annonc.2020.04.002 PMC7144604

[11] Alhalabi O Iyer S Subbiah V. Testing for COVID-19 in patients with cancer. *EClinicalMedicine.* (2020) 23:100374. 10.1016/j.eclinm.2020.100374 32368727 PMC7196399

[12] CDC *Interim Guidance for Discontinuation of Transmission-Based Precautions and Disposition of Hospitalized Patients with COVID-19.* Available online at: https://www.cdc.gov/coronavirus/2019-ncov/hcp/disposition-hospitalized-patients.html (2020) (accessed April 3, 2020).

[13] NHC Available online at: http://www.nhc.gov.cn/jkj/s3577/202003/0beb22634f8a4a48aecf405c289fc25e.shtml (2020) (accessed March 24, 2020).

[14] NHC Available online at: http://www.nhc.gov.cn/yzygj/s7653pd/202003/d4558d2cc35e44d5b9adba7c911e0b4c.shtml (2020) (accessed March 24, 2020).

[15] NHC Available online at: http://www.nhc.gov.cn/yzygj/s7653p/202003/46c9294a7dfe4cef80dc7f5912eb1989.shtml (2020) (accessed March 24, 2020).

[16] NHC Available online at: http://www.nhc.gov.cn/yzygj/s7653pd/202003/056b2ce9e13142e6a70ec08ef970f1e8.shtml (2020) (accessed March 24, 2020).

[17] NHC Available online at: http://www.nhc.gov.cn/yzygj/s7659/202003/c24669ab06324ad080ef7282cd26cf0a.shtml (2020) (accessed March 24, 2020).

[18] American College of Surgeons *COVID-19: Elective Case Triage Guidelines for Surgical Care.* Available online at: https://www.facs.org/covid-19/clinical-guidance/elective-case (2020) (accessed March 24, 2020).

[19] ASCO *COVID-19 Provider & Practice Information.* Available online at: https://www.asco.org/asco-coronavirus-information/provider-practice-preparedness-covid-19 (2020) (accessed April 4, 2020).

[20] COVID-19 Guidelines for Triage of Cancer Surgery Patients. Available online at: https://www.facs.org/covid-19/clinical-guidance/elective-case/cancer-surgery (2020) (accessed April 3, 2020).

[21] Ueda M Martins R Hendrie PC McDonnell T Crews J Wong T Managing cancer care during the COVID-19 pandemic: agility and collaboration toward a common goal. *J Natl Compr Canc Netw.* (2020) 18:1–4. 32197238 10.6004/jnccn.2020.7560

[22] ASCO *Care of Individuals with Cancer During COVID-19.* Available online at: https://www.asco.org/asco-coronavirus-information/care-individuals-cancer-during-covid-19 (2020) (accessed April 3, 2020).

[23] Cao B Wang Y Wen D Liu W Wang J Fan G. A trial of lopinavir-ritonavir in adults hospitalized with severe Covid-19. *N Engl J Med.* (2020) 382:1787–99. 32187464 10.1056/NEJMoa2001282 PMC7121492

[24] CHICTR Available online at: http://www.chictr.org.cn/uploads/documents/2020/04/02/51d7a6ef7df44aaabbe883c3789fd38b.xlsx (2020) (accessed April 3, 2020).

[25] ClinicalTrials.gov Available online at: https://www.clinicaltrials.gov/ct2/results?cond=COVID-19 (2020) (accessed April 3, 2020).

[26] Yang Y Islam MS Wang J Li Y Chen X. Traditional Chinese medicine in the treatment of patients infected with 2019-new Coronavirus (SARS-CoV-2): a review and perspective. *Int J Biol Sci.* (2020) 16:1708–17. 32226288 10.7150/ijbs.45538 PMC7098036

[27] CellPress Available online at: https://marlin-prod.literatumonline.com/pb-assets/journals/research/immunity/SARS-CoV-2%20vaccines%20status%20report.pdf (2020) (accessed April 3, 2020).

[28] FDA Available online at: http://fda.hubei.gov.cn/fbjd/tzgg/202003/t20200311_2178795.shtml (2020) (accessed April 3, 2020).

[29] FDA. *FDA Guidance on Conduct of Clinical Trials of Medical Products during COVID-19 Public Health EmergencyGuidance for Industry, Investigators, and Institutional Review Boards.* Available online at: https://www.fda.gov/media/136238/download (2020) (accessed April 3, 2020).

[30] Cancer.gov. *NCI Statement on Clinical Trials During COVID-19 Pandemic.* Available online at: https://www.cancer.gov/news-events/press-releases/2020/nci-statement-clinical-trials-during-covid-19 (2020) (accessed April 3, 2020).

[31] Porzio G Cortellini A Bruera E Verna L Ravoni G Peris F Home care for cancer patients during COVID-19 pandemic: the “double triage” protocol. *J Pain Symptom Manage.* (2020) 60:e5–7. 32240755 10.1016/j.jpainsymman.2020.03.021 PMC7165240

[32] Henson LA Maddocks M Evans C Davidson M Hichs S Higginson I. Palliative care and the management of common distressing symptoms in advanced cancer: pain, breathlessness, nausea and vomiting, and fatigue. *J Clin Oncol.* (2020) 38:905–14. 32023162 10.1200/JCO.19.00470 PMC7082153

[33] Zhang YZ Koopmans M Yuen KY Andersen K Perlman S Hogue B The novel coronavirus outbreak: what we know and what we don’t. *Cell.* (2020) 180:1034–6. 32078801 10.1016/j.cell.2020.02.027 PMC7154513

[34] CDC *Discontinuation of Isolation for Persons with COVID-19 Not in Healthcare Settings.* Available online at: https://www.cdc.gov/coronavirus/2019-ncov/hcp/ending-isolation.html (2020) (accessed April 3, 2020).

[35] Walls AC Park YJ Tortorici MA Wall A McGuire A Veesler D. Structure, function, and antigenicity of the SARS-CoV-2 spike glycoprotein. *Cell.* (2020) 181:281–92.e6. 32155444 10.1016/j.cell.2020.02.058 PMC7102599

[36] Xu X Yu C Qu J Zhang L Jiang S Huang D Imaging and clinical features of patients with 2019 novel coronavirus SARS-CoV-2. *Eur J Nucl Med Mol Imaging.* (2020) 47:1275–80. 32107577 10.1007/s00259-020-04735-9 PMC7080117

[37] CDC *Healthcare Facilities: Managing Operations During the COVID-19 Pandemic.* Available online at: https://www.cdc.gov/coronavirus/2019-ncov/healthcare-facilities/guidance-hcf.html (2020) (accessed April 3, 2020).

[38] American College of Surgeons *COVID-19 Guidelines for Triage of Thoracic Patients.* Available online at: https://www.facs.org/covid-19/clinical-guidance/elective-case/thoracic-cancer (2020) (accessed April 3, 2020).

[39] SSO *COVID-19 Resources.* Available online at: https://www.surgonc.org/resources/covid-19-resources (2020) (accessed April 3, 2020).

[40] Kimmig R Verheijen RHM Rudnicki M. Robot assisted surgery during the COVID-19 pandemic, especially for gynecological cancer: a statement of the Society of European Robotic Gynaecological Surgery (SERGS). *J Gynecol Oncol.* (2020) 31:e59. 10.3802/jgo.2020.31.e59 PMC7189073 32242340

[41] SSO *Resourcefor Management Options of Colorectal CancerDuring COVID-19.* (2020). Available online at: https://www.surgonc.org/wp-content/uploads/2020/03/Colorectal-Resource-during-COVID-19-3.30.20.pdf (accessed March 30, 2020)

[42] SSO *Resourcefor ManagementOptionsof GI and HPB CancersDuring COVID-19.* (2020). Available online at: https://www.surgonc.org/wp-content/uploads/2020/03/GI-and-HPB-Resource-during-COVID-19-3.30.20.pdf (accessed March 30, 2020)

[43] Wang Z Wang J He J. Active and effective measures for the care of patients with cancer during the COVID-19 spread in China. *JAMA Oncol.* (2020). 10.1001/jamaoncol.2020.1198 [Epub ahead of print], 32236504

[44] Wang Z Yang B Li Q Wen L Zhang R. Clinical features of 69 cases with Coronavirus disease 2019 in Wuhan, China. *Clin Infect Dis.* (2020) 71:769–77. 10.1093/cid/ciaa272 32176772 PMC7184452

[45] Cascella M Rajnik M Cuomo A Dulebohn S Napoli R. *Features, Evaluation and Treatment Coronavirus (COVID-19).* Treasure Island, FL: StatPearls Publishing. (2020). 32150360

[46] Cao J Tu WJ Cheng W Yu L Liu Y Hu X Clinical features and short-term outcomes of 102 patients with Corona Virus disease 2019 in Wuhan, China. *Clin Infect Dis.* (2020) 71:748–55. 32239127 10.1093/cid/ciaa243 PMC7184479

[47] Wang Y Wang Y Chen Y Qin Q. Unique epidemiological and clinical features of the emerging 2019 novel coronavirus pneumonia (COVID-19) implicate special control measures. *J Med Virol.* (2020) 92:568–76. 32134116 10.1002/jmv.25748 PMC7228347

[48] Guan WJ Ni Z Hu Y Liang W Ou C He J. Clinical characteristics of Coronavirus disease 2019 in China. *N Engl J Med.* (2020) 382:1708–20. 32109013 10.1056/NEJMoa2002032 PMC7092819

[49] Zhou F Yu T Du R Fan G Liu Y Liu Z Clinical course and risk factors for mortality of adult inpatients with COVID-19 in Wuhan, China: a retrospective cohort study. *Lancet.* (2020) 395:1054–62. 32171076 10.1016/S0140-6736(20)30566-3 PMC7270627

[50] Huang C Wang Y Li X Ren L Zhao J Hu Y Clinical features of patients infected with 2019 novel coronavirus in Wuhan, China. *Lancet.* (2020) 395:497–506. 31986264 10.1016/S0140-6736(20)30183-5 PMC7159299

[51] Chen N Zhou M Dong X Qu J Gong F Han Y Epidemiological and clinical characteristics of 99 cases of 2019 novel coronavirus pneumonia in Wuhan, China: a descriptive study. *Lancet.* (2020) 395:507–13. 32007143 10.1016/S0140-6736(20)30211-7 PMC7135076

[52] Wu P Duan F Luo C Liu Q Qu X Liang L Characteristics of ocular findings of patients with Coronavirus disease 2019 (COVID-19) in Hubei Province, China. *JAMA Ophthalmol.* (2020) 138:575–8. 32232433 10.1001/jamaophthalmol.2020.1291 PMC7110919

[53] Hoffmann M Kleine-Weber H Schroeder S Kruger N Herrler T Erichsen S SARS-CoV-2 cell entry depends on ACE2 and TMPRSS2 and is blocked by a clinically proven protease inhibitor. *Cell.* (2020) 181:271–80. 32142651 10.1016/j.cell.2020.02.052 PMC7102627

[54] Wu C Zheng S Chen Y Zheng M. Single-cell RNA expression profiling of ACE2, the putative receptor of Wuhan 2019-nCov. *medRxiv* [Preprint]. (2020). 10.1101/2020.02.11.20022228 medRxiv: 2020.02.11.20022228,

[55] Patel VB Zhong JC Grant MB Oudit G. Role of the ACE2/Angiotensin 1-7 Axis of the renin-angiotensin system in heart failure. *Circ Res.* (2016) 118:1313–26. 27081112 10.1161/CIRCRESAHA.116.307708 PMC4939482

[56] Imai Y Kuba K Rao S Huan Y Guo F Guan B Angiotensin-converting enzyme 2 protects from severe acute lung failure. *Nature.* (2005) 436:112–6. 16001071 10.1038/nature03712 PMC7094998

[57] Clerkin KJ Fried JA Raikhelkar J Sayer G Griffin JM Masoumi A COVID-19 and Cardiovascular disease. *Circulation.* (2020) 141:1648–55. 32200663 10.1161/CIRCULATIONAHA.120.046941

[58] Guo T Fan Y Chen M Wu X Zhang L He T Cardiovascular implications of fatal outcomes of patients with Coronavirus disease 2019 (COVID-19). *JAMA Cardiol.* (2020) 5:1–8. 10.1001/jamacardio.2020.1017 PMC7101506 32219356

[59] Dy GK Adjei AA. Understanding, recognizing, and managing toxicities of targeted anticancer therapies. *CA Cancer J Clin.* (2013) 63:249–79. 23716430 10.3322/caac.21184

[60] Wong SH Lui RN Sung JJ. Covid-19 and the digestive system. *J Gastroenterol Hepatol.* (2020). 10.1111/jgh.15047 [Epub ahead of print], 32215956

[61] Shelburne N Adhikari B Brell J Davis M Desvigne-Nickens P Freedman A Cancer treatment-related cardiotoxicity: current state of knowledge and future research priorities. *J Natl Cancer Inst.* (2014) 106:dju232. 10.1093/jnci/dju232 PMC4176042 25210198

[62] Curigliano G Cardinale D Dent S Criscitiello C Aseyev O Lenihan D Cardiotoxicity of anticancer treatments: epidemiology, detection, and management. *CA Cancer J Clin.* (2016) 66:309–25. 26919165 10.3322/caac.21341

[63] Herrmann J. Adverse cardiac effects of cancer therapies: cardiotoxicity and arrhythmia. *Nat Rev Cardiol.* (2020) 17:474–502. 10.1038/s41569-020-0348-1 32231332 PMC8782611

[64] Herrmann J. Common vascular toxicities of cancer therapies. *Cardiol Clin.* (2019) 37:365–84. 31587779 10.1016/j.ccl.2019.07.003 PMC6995033

[65] Qi WX Sun YJ Shen Z Yao Y. Risk of interstitial lung disease associated with EGFR-TKIs in advanced non-small-cell lung cancer: a meta-analysis of 24 phase III clinical trials. *J Chemother.* (2015) 27:40–51. 10.1179/1973947814y.0000000189 24724908

[66] Skeoch S Weatherley N Swift AJ Oldroyd A Johns C Havton C Drug-induced interstitial lung disease: a systematic review. *J Clin Med.* (2018) 7:356. 10.3390/jcm7100356 PMC6209877 30326612

[67] García-Gutiérrez V Hernández-Boluda JC. Tyrosine kinase inhibitors available for chronic myeloid leukemia: efficacy and safety. *Front Oncol.* (2019) 9:603. 10.3389/fonc.2019.00603 31334123 PMC6617580

[68] Loriot Y Perlemuter G Malka D Penault-Llorca F Boige V Deutsch E Drug insight: gastrointestinal and hepatic adverse effects of molecular-targeted agents in cancer therapy. *Nat Clin Pract Oncol.* (2008) 5: 268–78. 18349858 10.1038/ncponc1087

[69] Califano R Tariq N Compton S Fitzgerald D Harwood C Lal R Expert consensus on the management of adverse events from EGFR tyrosine kinase inhibitors in the UK. *Drugs.* (2015) 75:1335–48. 26187773 10.1007/s40265-015-0434-6 PMC4532717

[70] Liu W Tang F Fontanet A Zhan L Zhao Q Zhang P Long-term SARS coronavirus excretion from patient cohort, China. *Emerg Infect Dis.* (2004) 10:1841–3. 15504274 10.3201/eid1010.040297 PMC3323244

[71] Wang DY Salem JE Cohen JV Chandra S Menzer C Ye F Fatal toxic effects associated with immune checkpoint inhibitors: a systematic review and meta-analysis. *JAMA Oncol.* (2018) 4:1721–8. 30242316 10.1001/jamaoncol.2018.3923 PMC6440712

[72] Postow MA Sidlow R Hellmann MD. Immune-related adverse events associated with immune checkpoint blockade. *N Engl J Med.* (2018) 378:158–68. 29320654 10.1056/NEJMra1703481

[73] Samaan MA Pavlidis P Papa S Powell N Irving P. Gastrointestinal toxicity of immune checkpoint inhibitors: from mechanisms to management. *Nat Rev Gastroenterol Hepatol.* (2018) 15:222–34. 29512649 10.1038/nrgastro.2018.14

[74] Martins F Sykiotis GP Maillard M Fraga M Ribi C Kuntzer T New therapeutic perspectives to manage refractory immune checkpoint-related toxicities. *Lancet Oncol.* (2019) 20:e54–64. 30614479 10.1016/S1470-2045(18)30828-3

[75] Stroud CR Hegde A Cherry C Naqash A Sharma N Addepalli S Tocilizumab for the management of immune mediated adverse events secondary to PD-1 blockade. *J Oncol Pharm Pract.* (2019) 25:551–7. 29207939 10.1177/1078155217745144

[76] Mahmood SS Fradley MG Cohen JV Nohria A Reynolds K Heinzerling L Myocarditis in patients treated with immune checkpoint inhibitors. *J Am Coll Cardiol.* (2018) 71:1755–64. 29567210 10.1016/j.jacc.2018.02.037 PMC6196725

[77] Martins F Sykiotis GP Maillard M Fraga M Ribi C Kuntzer T New therapeutic perspectives to manage refractory immune checkpoint-related toxicities. *Lancet Oncol.* (2019) 20:e54–64. 30614479 10.1016/S1470-2045(18)30828-3

[78] CellPress Available online at: https://marlin-prod.literatumonline.com/pb-assets/journals/research/cell-host-microbe/chom_2283_s5.pdf (2020) (accessed April 3, 2020).

[79] Li G Fan Y Lai Y Han T Li Z Zhou P Coronavirus infections and immune responses. *J Med Virol.* (2020) 92:424–32. 31981224 10.1002/jmv.25685 PMC7166547

[80] Qin C Zhou L Hu Z Zhang S Yang S Tao Y Dysregulation of immune response in patients with COVID-19 in Wuhan, China. *Clin Infect Dis* (2020) 71:762–68. 32161940 10.1093/cid/ciaa248 PMC7108125

[81] Ling Y Xu SB Lin YX Tian D Zhu Z Dai F Persistence and clearance of viral RNA in 2019 novel coronavirus disease rehabilitation patients. *Chin Med J (Engl).* (2020) 133:1039–43. 32118639 10.1097/CM9.0000000000000774 PMC7147278

[82] Cui J Li F Shi ZL. Origin and evolution of pathogenic coronaviruses. *Nat Rev Microbiol.* (2019) 17:181–92. 30531947 10.1038/s41579-018-0118-9 PMC7097006

[83] Ye G Pan Z Pan Y Deng Q Chen L Li J Clinical characteristics of severe acute respiratory syndrome coronavirus 2 reactivation. *J Infect.* (2020) 80:e14–7. 10.1016/j.jinf.2020.03.001 PMC7102560 32171867

[84] Li YC Bai WZ Hashikawa T. The neuroinvasive potential of SARS-CoV2 may play a role in the respiratory failure of COVID-19 patients. *J Med Virol.* (2020) 92:552–5. 32104915 10.1002/jmv.25728 PMC7228394

[85] Park C Keam B Yoon SH Ock C Choi S Kim M Clinical insights on outcomes of corticosteroid administration in immune checkpoint inhibitor-induced pneumonitis by retrospective case series analysis. *ESMO Open.* (2019) 4:e000575. 10.1136/esmoopen-2019-000575 31803501 PMC6890388

[86] Naidoo J Wang X Woo KM Iyriboz T Halpenny D Cunningham J Pneumonitis in patients treated with anti-programmed death-1/programmed death ligand 1 therapy. *J Clin Oncol.* (2017) 35:709–17. 27646942 10.1200/JCO.2016.68.2005 PMC5559901

[87] Bai HX Hsieh B Xiong Z Halsey K Choi J Tran T Performance of radiologists in differentiating COVID-19 from viral pneumonia on chest CT. *Radiology.* (2020) 296:E46–54. 32155105 10.1148/radiol.2020200823 PMC7233414

[88] Ai T Yang Z Hou H Zhan C Chen C Lv W Correlation of chest CT and RT-PCR testing in Coronavirus disease 2019 (COVID-19) in China: a report of 1014 cases. *Radiology.* (2020) 296:E32–40. 32101510 10.1148/radiol.2020200642 PMC7233399

[89] Shi H Han X Jiang N Cao Y Alwalid O Gu J Radiological findings from 81 patients with COVID-19 pneumonia in Wuhan, China: a descriptive study. *Lancet Infect Dis.* (2020) 20:425–34. 32105637 10.1016/S1473-3099(20)30086-4 PMC7159053

[90] Fang Y Zhang H Xu Y Xie J Pang P Ji W CT manifestations of two cases of 2019 novel Coronavirus (2019-nCoV) pneumonia. *Radiology.* (2020) 295:208–9. 32031481 10.1148/radiol.2020200280 PMC7233358

[91] Xu Y Liu H Hu K Wang M. Clinical management of lung cancer patients during the outbreak of 2019 novel Coronavirus disease (COVID-19). *Zhongguo Fei Ai Za Zhi.* (2020) 23:136–41. 32077441 10.3779/j.issn.1009-3419.2020.03.02 PMC7118333

[92] Jazieh AR Al Hadab A Al Olayan A AlHejazi A AlSafi F AlQarni A Managing oncology services during a major Coronavirus outbreak: lessons from the Saudi Arabia experience. *JCO Glob Oncol.* (2020) 6:518–24. 32216653 10.1200/GO.20.00063 PMC7124946

[93] Turner NC Ro J André F Loi S Verma S Iwata H Palbociclib in hormone-receptor-positive advanced breast cancer. *N Engl J Med.* (2015) 373:209–19. 26030518 10.1056/NEJMoa1505270

[94] Spring LM Wander SA Andre F Moy B Turner N Bardia A Cyclin-dependent kinase 4 and 6 inhibitors for hormone receptor-positive breast cancer: past, present, and future. *Lancet.* (2020) 395:817–27. 32145796 10.1016/S0140-6736(20)30165-3

[95] Rodriguez-Ruiz ME Vitale I Harrington KJ Melero I Galluzzi L. Immunological impact of cell death signaling driven by radiation on the tumor microenvironment. *Nat Immunol.* (2020) 21:120–34. 31873291 10.1038/s41590-019-0561-4

[96] Ngwa W Irabor OC Schoenfeld JD Hesser J Demaria S Formenti S Using immunotherapy to boost the abscopal effect. *Nat Rev Cancer.* (2018) 18:313–22. 29449659 10.1038/nrc.2018.6 PMC5912991

[97] Filippi AR Russi E Magrini S Corvo R. Covid-19 outbreak in Northern Italy: first practical indications for radiotherapy departments. *Int J Radiat Oncol Biol Phys.* (2020) 107:597–9. 32199941 10.1016/j.ijrobp.2020.03.007 PMC7141469

[98] Wang Y Dong C Hu Y Li C Ren Q Zhang X Temporal changes of CT findings in 90 patients with COVID-19 pneumonia: a longitudinal study. *Radiology.* (2020) 296:E55–64. 32191587 10.1148/radiol.2020200843 PMC7233482

[99] Coles CE Aristei C Bliss J Boersma L Brunt A Chatterjee S International guidelines on radiation therapy for breast cancer during the COVID-19 pandemic. *Clin Oncol.* (2020) 32:279–81. 10.1016/j.clon.2020.03.006 PMC7270774 32241520

[100] Karam SD Raben D. Radioimmunotherapy for the treatment of head and neck cancer. *Lancet Oncol.* (2019) 20:e404–16. 31364593 10.1016/S1470-2045(19)30306-7

[101] Inoue A Saijo Y Maemondo M Gomi K Tokue Y Kimura Y Severe acute interstitial pneumonia and gefitinib. *Lancet.* (2003) 361:137–9. 12531582 10.1016/S0140-6736(03)12190-3

[102] May P Normand C Morrison RS. Economics of palliative care for cancer: interpreting current evidence, mapping future priorities for research. *J Clin Oncol.* (2020) 38:980–6. 32023166 10.1200/JCO.18.02294

[103] Hanna TP Evans GA Booth CM. Cancer, COVID-19 and the precautionary principle: prioritizing treatment during a global pandemic. *Nat Rev Clin Oncol.* (2020) 17:268–70. 32242095 10.1038/s41571-020-0362-6 PMC7117554

[104] UICC *Coronavirus and Cancer Care: Planning, Informing, Assisting and Giving Hope.* Available online at: https://www.uicc.org/news/coronavirus-and-cancer-care-planning-informing-assisting-and-giving-hope (2020) (accessed April 3, 2020).

[105] Xu X Yu C Qu J Zhang L Jiang S Huang D Imaging and clinical features of patients with 2019 novel coronavirus SARS-CoV-2. *Eur J Nucl Med Mol Imaging.* (2020) 47:1275–1280. 10.1007/s00259-020-04735-9 32107577 PMC7080117

